# Therapeutic Effects of Pregnancy on a Chronic Skin Ulcer

**Published:** 2013-06-13

**Authors:** Luca Lancerotto, Gianluigi Lago, Elena Pescarini, Franco Bassetto, Vincenzo Vindigni

**Affiliations:** Clinic of Plastic Surgery, University of Padova, Padova, Italy

## Abstract

**Objectives:** Sex hormones strongly influence skin physiology and evidences suggest wound healing as well. Estrogens establish a prohealing setting, and androgens play an antagonist role. **Methods and Results:** We present the case of a young woman in whom pregnancy with its associated hormonal shifts allowed the spontaneous healing of a chronic wound on the left leg with bone exposure, which had followed a car accident and had resisted medical and surgical treatments for 5 years. **Discussion:** Systemic or topical estrogens may offer alternative options in the therapy of chronic wounds, in particular in the elderly adults who have physiologically reduced estrogens levels.

Chronic skin ulcers are a major social burden in an increasingly elderly population, which hamper patients’ life and represent high costs for health care systems. Incapacity to heal is determined by a multitude of local and systemic factors, such as peripheral arteriopathy, venous insufficiency, diabetes, nutritional deficits, and infection. Alone or, more often, in combination, these exacerbate inflammatory phenomena and frustrate the proliferative phase of wound healing by an ultimate action on microenvironment and cells. The contribution of hormonal unbalances is often less appreciated. However, hormones deeply influence cells activity. Glucocorticoids are powerful inhibitors on almost all cell types, while a lack of thyroid hormones impairs fibroblasts function.^1^ Sex hormones are given limited consideration as far as wound healing is concerned but are known to play a key role in maintaining tissues trophism.^2^ We present a case in which pregnancy and its major hormonal fluctuations allowed the healing of a nonresponding chronic wound with bone exposure.

## CASE REPORT

**Figure 1:**
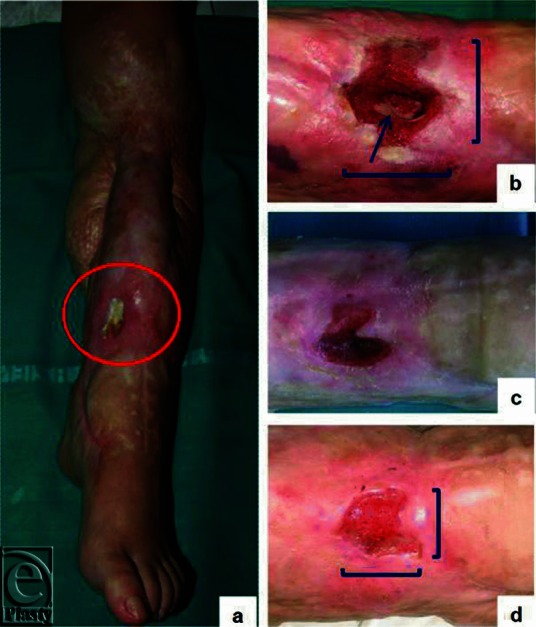
(*a*) Posttraumatic chronic wound on the left leg with tibial exposure upon admittance to our wound care center, (*b*) after surgical debridement (arrow: exposed bone), (*c*) the gap started to decrease in size with pregnancy, and (*d*) superficialized well-granulating wound without bone exposure by the sixth month of pregnancy.

A 30-year-old woman consulted us for a chronic skin ulcer with extensive soft tissues loss and tibial exposure at the left leg, without fever or other signs of acute infection (Fig 1a). She had suffered a complete degloving of the leg with multiple bone fractures in a car accident 5 years before. Treatment had been successful in saving the limb, but a nonhealing wound with tibial exposure developed. An angiographic study showed patency of the peroneal artery only, the reason why recourse to free flaps had been excluded. The persistent lesion had been repetitively treated with adipose tissue grafts, negative pressure therapy, and other advanced biointeractive skin dressings without any improvement. The burden of the wound even led the patient to consider amputation as an option. We performed a surgical debridement (Fig 1b) as part of a multiple-step surgical plan, but then the patient refused further surgery and was directed to outpatient clinic follow-up. Dressing changes were performed with standard saline solution alone for 5 months, with no substantial improvement. Then the patient became pregnant, and surprisingly the lesion started to heal (Fig 1c). By the sixth month of pregnancy, granulation tissue completely covered the bone and filled the ulcer, which displayed significantly reduced diameters (Fig 1d). The patient moved back to her home country for delivery and was lost to follow-up.

## DISCUSSION

The influence of sex hormones on wound healing only recently has been gaining higher attention. Chronic wounds mostly occur in the elderly adults,^3^ whose skin is thinner, more fragile, and has reduced function as a result of cell senescence.^4^ With increasing age, the endocrine system, in particular the adrenal axis, undergoes substantial change. Clinically, androgens are more elevated in men and maintained with aging; estrogens decrease with age, particularly in women after menopause. Estrogens and androgens play antagonist roles. Estrogens enhance antioxidant pathways and upregulate telomerases, contrasting cell senescence.^4^ With respect to wound healing, estrogens dampen inflammation, stimulate keratinocytes and dermal fibroblasts activity, and enhance angiogenesis;^5^ androgens have proinflammatory effects, and treatment with antagonist flutamide was reported to accelerate repair.^6^ Male sex has been suggested as predisposing factor to venous ulceration, while hormone replacement therapy reduces the risk of venous ulceration in elderly women.^5^^,^^7^ Indeed, patients with a history of chronic wounds have reduced levels of dehydroepiandrosterone, a precursor locally converted to estrogen, compared with age-matched controls, and local dehydroepiandrosterone supplementation accelerated wound healing in estrogen-deficient mice.^8^ Topical and systemic treatments with estrogens were reported to improve healing in men and in elderly women; topical estrogens stimulate contraction, increase the rate of re-epithelization and collagen deposition; similar effects were observed in postmenopausal women treated with hormone replacement therapy.^7^ The effects of androgens are less understood, but the negative impact on wound healing is well-accepted. In healthy men, elevated testosterone levels correlated with delayed healing of punch biopsies,^6^ and elderly men suffering of venous ulcerations displayed significantly higher levels of di-hydrotestosterone than age-matched controls.^9^

Pregnancy is a peculiar condition in which the sex-hormone balance is strongly altered. A sudden shift toward estrogens and progesterone occurs, estradiol increasing 5-fold in the first trimester and 32-fold by week 40, while androgens remain substantially stable and the free androgen index markedly decreases (5-fold between week 5 and week 21).^10^ The main source of estrogens during pregnancy is the placenta, where the precursors are aromatized. Levels of circulating dehydroepiandrosterone, indicated as candidate for sex steroids replacement therapy in wound healing, decrease during pregnancy because of increased clearance by conversion. This is in agreement with studies suggesting that its beneficial action on wounds is dependent on local conversion in estrogens.^8^ A systemic healing-conductive setting is thus established, that likely explains the unexpected healing we observed of an ulcer with bone exposure that had not responded to any treatment for years.

Other pregnancy-associated elements may also have played a role in the induction of healing seen in our patient, for example, changes in blood flow, liquids retention, or availability of metabolites affecting the wound environment, as well as parallel alternative signal pathways.

On the contrary, given the cumulating evidences, the role of sex hormones in tissue repair seems to merit further investigation; our clinical observation suggests a possible major impact of the introduction of hormonal therapy as skin lesions medical treatment.
